# Modified-release of encapsulated bioactive compounds from annatto seeds produced by optimized ionic gelation techniques

**DOI:** 10.1038/s41598-020-80119-1

**Published:** 2021-01-14

**Authors:** Ana María Naranjo-Durán, Julián Quintero-Quiroz, John Rojas-Camargo, Gelmy Luz Ciro-Gómez

**Affiliations:** College of Pharmaceutical and Food Sciences, University of Antioquia, Street 67, 53-108 Medellin, Colombia

**Keywords:** Chemical engineering, Drug delivery

## Abstract

To compare the encapsulation of annatto extract by external gelation (EG) and internal gelation (IG) and to maximize process yield (% Y), two central composite designs were proposed. Calcium chloride (CaCl_2_) concentration (0.3–3.5%), alginate to gelling solution ratio (1:2–1:6); acetic acid (CH_3_COOH) concentration (0.2–5.0%) and alginate to gelling solution ratio (1:2–1:6) were taken as independent variables for EG and IG respectively. Release studies were conducted under different conditions; morphology, particle size, the encapsulation efficiency (EE), and release mechanism were evaluated under optimized conditions. The optimized EG conditions were 0.3% CaCl_2_ and 1:1.2 alginate to gelling solution ratio, whereas a 0.3% CH_3_COOH and 1:5 alginate to gelling solution ratio were optimized conditions for IG. When 20% extract was employed, the highest EE was achieved, and the largest release was obtained at a pH 6.5 buffer. The Peppas–Sahlin model presented the best fit to experimental data. Polyphenol release was driven by diffusion, whereas bixin showed anomalous release. These results are promising for application as modulated release agents in food matrices.

## Introduction

*Bixa orellana* L. (annatto) is a native shrub from Latin America, it is widely cultivated due to the high content of carotenoids such as bixin in the seeds. This compound is a natural dye that works as an alternative substitute for synthetic dyes. In addition, the aqueous extract from the seeds has also been revealed to have antioxidant and antimicrobial properties attributed to their high content of carotenoid and polyphenol compoundds^1,2^. About 80% of carotenoids correspond to bixin (methyl hydrogen 9′-*cis*-6, 6′-diapocarotene-6,6′-dioate)^3^. This compound has nine conjugated double bonds and two carboxylic groups responsible for its ability to trap reactive oxygen and nitrogen species, along with free radicals^4^. The main polyphenol compounds found in the extract are catechin, chlorogenic acid, chrysine, butein, hypoaletin, licochalcona, and xanthoangelol^5^. The compounds can limit the action of free radicals and inhibit the growth of microorganisms in food products.

Furthermore, they can form extracellular complexes with cell wall proteins^3,6^. However, the major drawback of annatto extracts is its low stability, leading to the rapid degradation of the bioactive compounds (BC), resulting in poor performance if applied in food matrixes. The encapsulation technique has been shown to improve the effectiveness of preventing these compounds' degradation and could also achieve desirable effects like controlled delivery and extending shelf life to improve this extract's use for several food and pharmaceutical applications^4^.

Ionic gelation is one of the most straightforward and inexpensive techniques for encapsulating bioactive compounds since it does not require high temperatures or solvents; thus, it is especially useful for heat-sensitive compounds. There are two major types of ionic gelation; the first is external gelation (EG), and the second is internal gelation (IG). In EG, a sodium alginate solution containing the extract is extruded into a calcium chloride solution, and the capsule is formed when the Ca^2+^ ions diffuse from the salt solution toward the polymer and the BC. IG instead occurs when a solution containing alginate, extract, and CaCO_3_ is extruded into a medium composed of acidified oil. The reaction starts when the Ca^2+^ ions solubilize in the acidified medium, react with the alginate through ionic interactions, and form hydrogels, which entrap the BC. The significant difference between these two mechanisms is based on the reaction kinetics, which affects the wall structure, encapsulation efficiency, and BC release rate^7^. Some authors have studied the different properties between particles obtained by these two gelation mechanisms. Lupo et al. in 2015^7^ reported that the particles obtained by EG are smaller and harder than particles by IG. These characteristics are attributed to gelation kinetics on the surface, due to the more rigid layer it forms when calcium migrates from outside the drops. Instead, the particles obtained by IG are more homogeneous and smoother on their surface because gelation occurs from inside the drop. In the same way, Leong et al. in 2016^8^ concluded that the internal and external gelation mechanisms provide different particle sizes and morphological properties. However, no study was found comparing these processes already optimized using the response surface methodology and the evaluation of bixin and polyphenols release kinetic from the beads obtained by both encapsulation technics IG and EG. Therefore, this study aimed to compare the bioactive compounds' physicochemical properties and release rate from annatto extract encapsulated by IG and EG's optimized process.

## Materials and methods

### Production and characterization of the extract obtained from annatto seeds

The extract was obtained by leaching using ethanol as a solvent. The conditions adopted were a pH of 4.0, a 1:5 seed-to-solvent ratio, a temperature of 25 °C and constant stirring for 48 h^8^. The extract thus obtained was filtered and the liquid phase concentrated at 60 mbar and 35 °C (R-114, Rotavapor^®^, B-CHI). This solid was combined with the solid residue using a high shear homogenizer (D-500, SUCCESS TECHNIQUE) followed by freeze-drying and storing at 4 °C until further use.

### Experimental conditions

The optimal EG and IG conditions were determined by a surface response experimental design (RSM), using the Design Expert^®^ software (Vs. 8.0.6, Stat-Ease, USA). The calcium chloride concentration [CaCl_2_: 0.3–3.5% (w/v)] and gelling solution ratio (A:GS 1:2–1:6) were taken as independent variables for EG. On the other hand, for IG were glacial acetic acid concentration ([CH_3_COOH]: 0.2–0.5%), and alginate:gelling solution ratio (A:GS 1:2–1:6). In both methods, the process yield Y % (w/w)) was taken as the response variable and it was determined as follows:

1 $$Y = \frac{Experimental \; quantity \;of \;obtained \;beads}{{Theoretical\; quantity \;of \;obtained \;beads}} \times 100 \%$$

1% (w/v) sodium alginate solution was poured into a CaCl_2_ as a gelling solution using a 21-gauge needle syringe to obtain beads by EG. The solution was then stirred at 700 rpm with a magnet (Corning™ PC-420 d) for 15 min. The resulting beads were filtered and washed with distilled water^7^. Likewise, beads were produced by IG pouring a mixture of 1% sodium alginate solution and 0.35% (w/v) CaCO_3_ into a mixture of sunflower oil and acetic acid gelling solution. The mixture was then stirred at 700 rpm for 1 h. The resulting beads were separated from the acidic oil by centrifugation at 600 rpm for 10 min (centrifuge Z 206 A HERMLE) and degreased with petroleum ether. The yield of both methods was then determined (Eq. 1). The experimental data obtained for EG and IG were adjusted to polynomial models by multiple regressions. The coefficients of determination (r^2^) and the adjusted coefficient of determination (r^2^-adj) were used as the regression model's adjustment parameters. The statistical significance of the independent variables and the models were obtained by analyzing variance (ANOVA) using the LSD-Fisher test with a 95% confidence level. The response optimization was conducted in order to maximize yield for both techniques^9^.

### Encapsulation efficiency of annatto extract by the optimized EG and IG techniques

This was calculated indirectly from the alginate beads containing extract concentrations of 5, 10, and 20% (v/v), following the method described by Chan (2011)^10^ with minor modifications. Briefly, the content of bixin and total polyphenol compounds in the alginate dispersions was determined after the encapsulation process. The encapsulation efficiency of the extract was determined using Eq. (2). The experiments were conducted in triplicate.

2 $$\% EE = \frac{{m_{a} - m_{b} }}{{m_{a} }} \times 100$$

where, m_a_ and m_b_ correspond to the initial amount of BC and the non-encapsulated amount of these materials, respectively.

#### Quantification of total polyphenol compounds

The total polyphenol content was determined by spectrophotometry using the Folin–Ciocalteu method described by Nunes et al.^11^. Briefly, 30 μL of the sample was poured into 96-well microplates followed by 150 μL of Folin–ciocalteu reagent (0.2 N) and 120 μL of Na_2_CO_3_ (7.5%, w/v). The microplates were incubated in darkness for 15 min at 45 °C, followed by incubation at 25 °C for 30 min. Subsequently, the sample absorbance was measured at 765 nm using a spectrophotometer (Multiskan GO, Thermo Fisher Scientific). All measurements were taken in triplicate and expressed as mg equivalent of Gallic acid per mL (0–216 μg/mL; r^2^ = 0.999).

#### Quantification of bixin

75 mg of sample was mixed with 1.5 mL of dimethylsulfoxide, followed by dilution with acetone to obtain an absorbance value ≤ 0.15 at 487 nm employing a UV/VIS spectrophotometer (1700, Shimadzu Europe^®^). The concentration of bixin in the sample was determined using the following expression:^12^

3 $${\text{Bixin}}\;({\text{mg/mL}}) = \frac{A \times 1000 \times V }{{A_{{1\;{\text{cm}}}}^{1\% } \times 100}}$$

where: $A_{{1{\text{cm}}}}^{{1{\text{\% }}}}$: specific absorptivity coefficient of bixin in acetone (3090 g/100 mL)^−1^ × 1 cm^−1^; A: sample absorbance and V: dilution volume (mL)^13,14^.

### Morphology and particle size of the beads

These were determined using a Scanning Electron Microscope (SEM, Joel 6490LV, Peabody, MA, USA) at an accelerated voltage of 20 kV. The beads were fixed to aluminum sample holders and coated with a 5 nm gold layer using a vacuum chamber (Desk IV, Denton Vacuum, Moorestown, NJ, USA)^15^. The particle size was measured by digital image analysis using the method described by Londoño and Rojas^16^, with minor modifications. The microphotographs were taken with a digital camera. Around 1000 particles were randomly selected per microphotograph and their projected area, aspect ratio (AR) and sphericity were determined using the ImageJ^®^ software.

### Infrared transmission spectroscopy (FT-IR) characterization

This was conducted according to the method described by Belscak-Cvitanovic et al*.*^17^. The samples were mixed with KBr in an agate mortar and compressed into pellets using a single tablet press. Spectra were taken on a FT-IR spectrophotometer (IRAffinity-1 SHIMADZU) in a range between 4000 and 400 cm^−1^, 16 scans with a resolution of 4 cm^−1^.

### Release studies of polyphenol compounds and bixin from the beads

The effect of pH (3.0, 6.5 and 10), ionic strength (0 and 100 mM), thermal treatment (60 and 90 °C), and surfactants (anionic and non-ionic) were studied. The pHs of 3.0 and 6.5 were used sodium phosphate buffer, whereas the tris buffer was pH to 10. The ionic strength was adjusted with NaCl solutions at 0 and 100 mM. The surfactant effect was studied with a 5% SDS and a 2% of tween 80. The thermal treatment was studied at 60 °C for 30 min and 90 °C for 10 min^18,19^.

Approximately, 1 g of beads were poured into a polypropylene bag (1 cm^2^), and placed into 5 mL of the solutions maintained at the conditions described previously and stirred at 210 rpm employing a shaker (HZQ-120H, Thermo) at 25 °C. 1 mL Aliquots were taken at 15, 30, 45, 60, 90, 120, 150, 180, 210, 240, 300 and 360 min and at 12 h and 24 h. The respective solution immediately replaced the respective volume taken. The concentration of total polyphenol compounds and bixin was then calculated using a calibration curve conducted with the same media. All measurements were performed in triplicate^20^.

The release kinetics was studied according to the method described by Yu et al*.* with minor modifications^21^. 100 mg of beads were poured into 15 mL was stirring at 210 rpm for 48 h. Subsequently, aliquots were taken at 1, 2, 3, 4, 5, 6, 12, 24, and 48 h the content of polyphenol compounds and bixin was determined. The release kinetics of the BC (f vs t) was plotted until reaching a released fraction of 0.6. The experimental data were adjusted to the kinetic Peppas, Peppas–Burst, and Peppas–Sahlin models, which showed the best fit ^21,22^ (Eqs. 4, 5, and 6):

4 $$f = k \times t^{n}$$

5 $$f = k \times t^{n} + \alpha$$

6 $$f = (k_{1} \times t^{m} ) + (k_{2} \times t^{2m} )$$

where: f, k, n, k_1_, k_2_ and m corresponds to the fraction of compound released, the system structural and geometric characteristics, release mechanism, Fickian diffusion, relaxation mechanism and diffusive geometric coefficient, respectively.

### Statistical analysis

The results were expressed as mean ± standard deviation (SD) of three replicate. The experimental designs, analysis of variance, and mathematical models were conducted using the STATGRAPHICS CENTURION^®^ software. Comparisons of the means were made with the Fisher LSD test with a 95% confidence level.

## Results

### Optimization of the external (EG) and internal (IG) ionic gelation mechanisms

The numerical optimization and the grid search within the contour plots were employed to select the optimal region that presented the maximized yield. Yield varied from 22.89 to 97.40% for the EG process (Table 1). Therefore, the optimized conditions were 0.3% to CaCl_2_ (equivalent to 0.0024 moles of Ca^2+^/g_alginate_) and ratio (A:GS) of 1:1.2. The optimized parameters were validated experimentally by conducting a checkpoint run with a resulting yield of 88.06 ± 0.55% with a relative bias of − 0.12. On the other hand, the IG encapsulation process rendered the highest yield when 0.30% CH_3_COOH and a 1:5 (A:O) ratio were employed, resulting in a yield of 56.51 ± 3.18. Furthermore, a validation run rendered a 56.51 ± 3.18% yield with a relative bias of 11.91%. No studies show the use of IG techniques for the encapsulation of these types of extracts; perhaps the resulting low yields are responsible for this.

**Table 1:** Experimental design matrix for optimization of yield.

Run	Rotary composite central factorial design for EG			Face-centered composite central factorial design for IG		
	[CaCl_2_] (%)	A:GS 1:x	Y (%)	[CH_3_COOH] (%)	A:GS 1:x	Y (%)
1	1.9	4.0	38.47	0.3	0.2	0.00
2	0.8	2.0	65.30	0.3	3.0	48.87
3	1.9	4.0	37.64	0.1	1.0	0.00
4	3.0	2.0	49.18	0.3	3.0	53.18
5	3.5	4.0	30.27	0.1	5.0	70.59
6	1.9	4.0	44.36	0.1	5.0	70.83
7	1.9	6.8	26.07	0.3	0.2	0.00
8	0.8	6.0	44.24	0.3	5.8	49.72
9	3.0	6.0	23.62	0.5	5.0	54.87
10	0.3	4.0	73.81	0.1	1.0	0.00
11	1.9	1.2	64.04	0.6	3.0	50.64
12	1.9	4.0	44.88	0.5	1.0	34.41
13	0.8	2.0	70.21	0.3	5.8	55.02
14	1.9	4.0	39.24	0.3	3.0	37.98
15	3.0	2.0	54.66	0.3	3.0	58.84
16	3.5	4.0	38.34	0.6	3.0	52.25
17	1.9	4.0	50.00	0.5	5.0	54.05
18	1.9	6.8	35.35	0.3	3.0	53.67
19	0.8	6.0	50.04	0.02	3.0	0.00
20	3.0	6.0	22.89	0.3	3.0	55.91
21	0.3	4.0	89.94	0.5	1.0	31.62
22	1.9	1.2	83.73	0.02	3.0	0.00
23	1.9	4.0	44.88	N.A	N.A	N.A
24	0.8	2.0	81.97	N.A	N.A	N.A
25	1.9	4.0	52.91	N.A	N.A	N.A
26	3.0	2.0	59.19	N.A	N.A	N.A
27	3.5	4.0	28.65	N.A	N.A	N.A
28	1.9	4.0	51.39	N.A	N.A	N.A
29	1.9	6.8	35.79	N.A	N.A	N.A
30	0.8	6.0	53.60	N.A	N.A	N.A
31	3.0	6.0	30.11	N.A	N.A	N.A
32	0.3	4.0	85.56	N.A	N.A	N.A
33	1.9	1.2	97.40	N.A	N.A	N.A
Predicted value	0.30	1:1.2	100	0.3	1:5	63.24
Experimental value	0.30	1:1.2	88.06 ± 0.55	0.3	1:5	56.51 ± 3.18

*[CaCl*_*2*_*] (%)* calcium chloride concentration, *A:GS (1:X)* alginate: gelling solution ratio, *[CH*_*33*_*COOH] (%)* acetic acid concentration, *Y (%)* process yield percentage.

The analysis of variance (ANOVA) of the experimental design applied to the EG process (Table 2) shows the significance of the linear and quadratic terms (*p* < 0.05) and explain 82.1% of the yield variability. However, no statistically significant differences were found between the interaction factors (*p* = 0.6349). Likewise, the ANOVA table for IG indicates that the model, the factors evaluated in their linear and quadratic terms, and their linear interaction were statistically significant (*p* < 0.05) and explained 82.33% of the yield variability.

**Table 2:** ANOVA Table for the ionic gelation optimization process.

EG		IG	
	*p *value		*p *value
A-[CaCl_2_]	< 0.0001	A-[CH_3_COOH]	0.0005
B-A:GS	< 0.0001	B-A:O	< 0.0001
AB	0.6349	AB	0.0049
A^2^	0.0295	A^2^	0.0077
B^2^	0.0423	B^2^	0.0099
r^2^	0.8616	r^2^	0.8653
r^2^-adjusted	0.8212	r^2^-adjusted	0.8233

*EG* external gelation, *IG* internal gelation, *[CaCl*_*2*_*] (%)* calcium chloride concentration, *A:GS (1:X)* alginate: gelling solution ratio, *[CH*_*3*_*COOH] (%)* acetic acid concentration.

**Figure 1:**
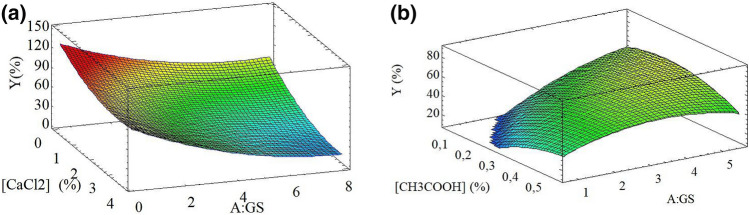
Responses surfaces plots for (%Y) resulted from (**a**) EG and (**b**) IG.

Equations (7) and (8) show the polynomial equation for yield as a function of the factors evaluated, and Fig. 1 depicts the surface response plots showing the effect of the independent variables on yield by EG and IG, respectively. In panel A, yield increased as the CaCl_2_ concentration and ratio A:GS decreased. Therefore, panel B shows that as the A:GS ratio increased, yield increased, especially at low acetic acid levels. Also, at 63.24% yield, there is an inflection point demonstrating the quadratic effect with the increase of acetic acid.

7 $$Y \;(\% ) = 126.991 - 25.491A - 15.295B + 3.920 A^{2} - 0.559AB + 1.099B^{2}$$

8 $$Y\; (\% ) = - 66.042 + 283.564A + 33.925B - 30.791AB - 224.953A^{2} - 2.373B^{2}$$

### Encapsulation efficiency of annatto extract conducted under the optimized conditions

**Figure 2:**
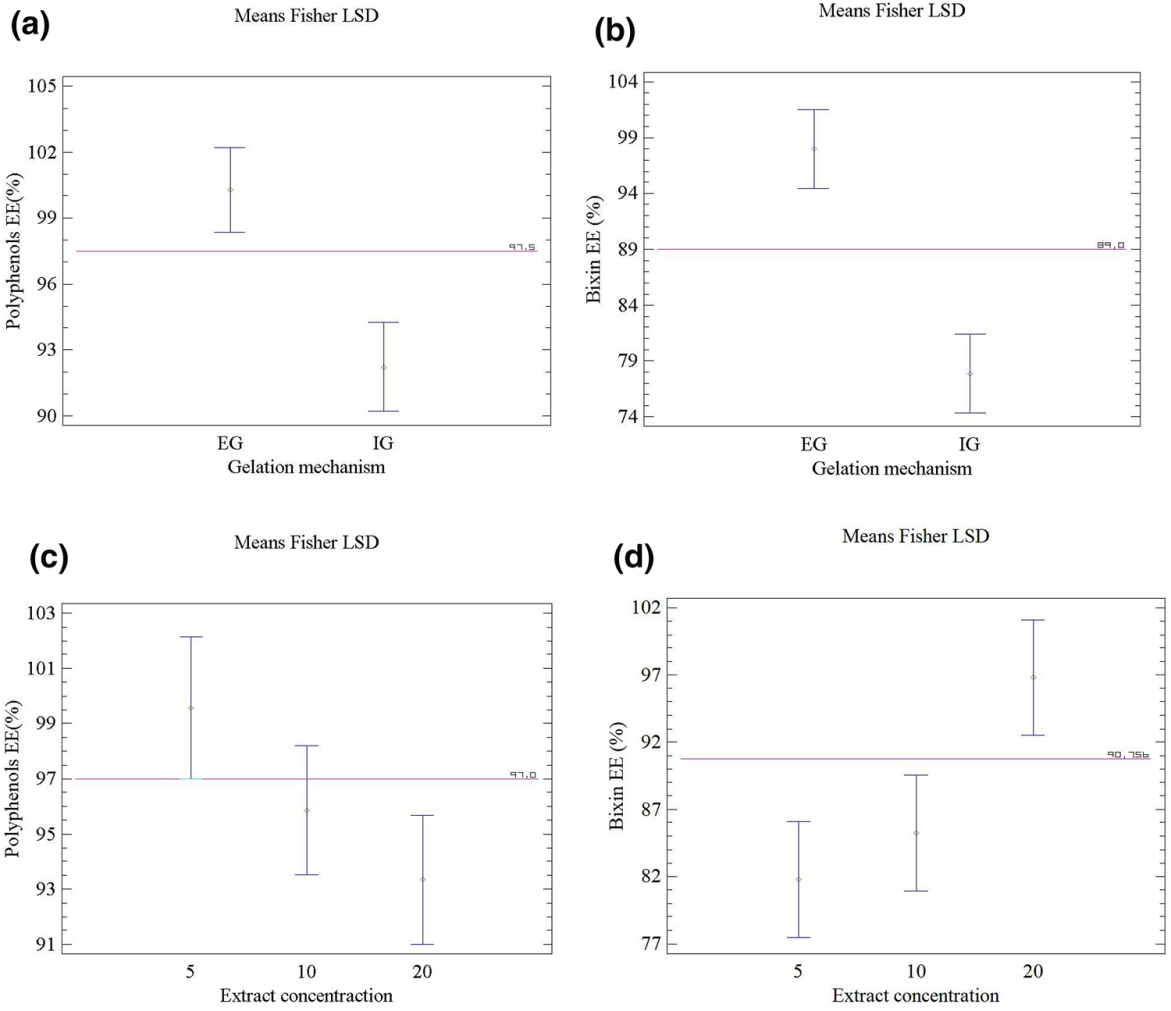
Fisher's LSD test with 95% confidence for: (**a**) EE for polyphenols compounds with respect to gelation type; (**b**) EE for bixin with respect to gelation type; (**c**) EE for polyphenol compounds with respect to the extract concentration; (**d**) EE of bixin with respect to the extract concentration.

A statistically significant effect was found for both factors in bixin (*p* < 0.05). Conversely, the EE of polyphenol compounds showed the gelation type as the only statistically significant factor (*p* = 0.0007). Fisher's LSD test comparing the EE concerning the gelation type is shown in Fig. 2a,b. The EE of polyphenol compounds and bixin was much higher when the extract was encapsulated by the EG rather than IG (*p* < 0.05). Likewise, the EE also varied concerning the extract (Fig. 2c,d). There were no statistically significant differences between the EE of polyphenol compounds for the extract at concentrations from 5 to 20%. Conversely, the EE of bixin (Fig. 2d) reached 100% when a 20% extract was employed (*p* < 0.005)*.*

### Particle size and morphology

**Figure 3:**
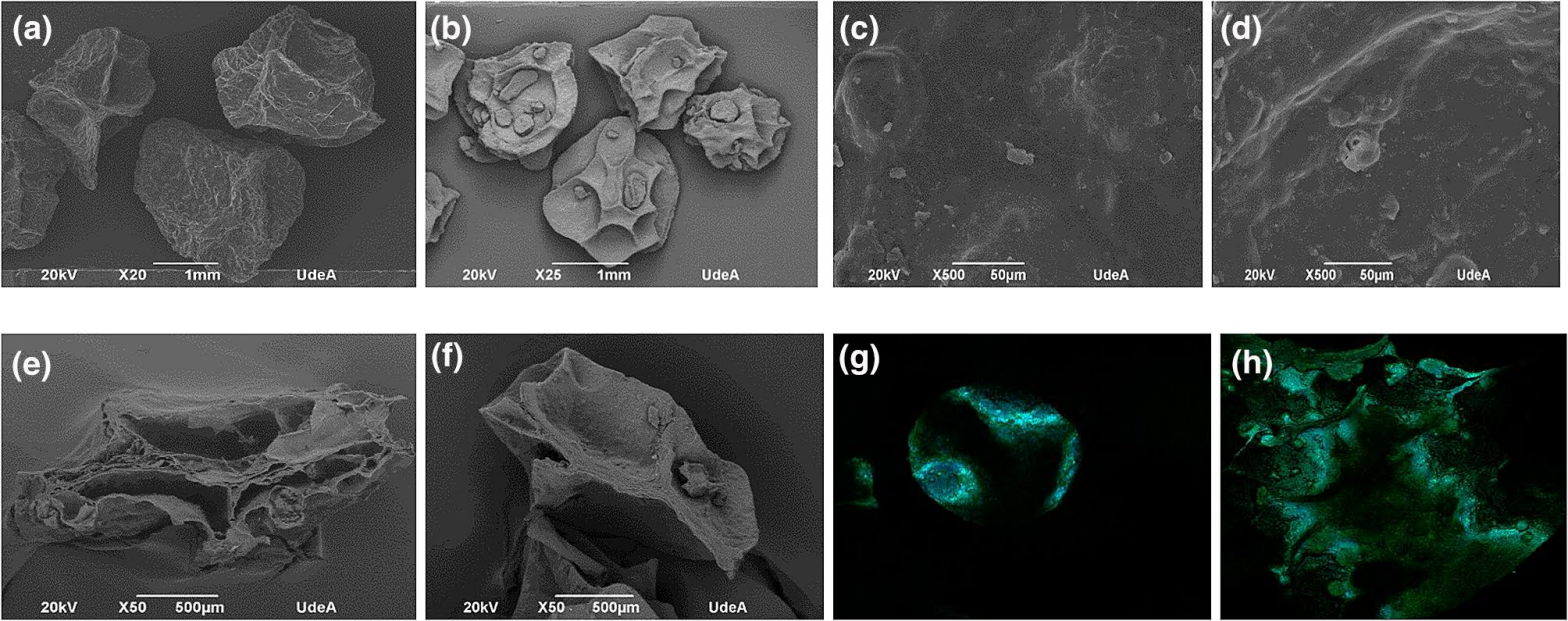
SEM micrographs for beads obtained by EG and IG. (**a**) EG beads at 20 ×; (**b**) IG beads at 25 ×; (**c**) surface obtained by EG at 500 ×; (**d**) surface obtained by IG at 500 ×; (**e**) Cross-sectional view obtained by EG at 50 ×; (**f**) cross-sectional view obtained by IG at 50 ×, (**g**) confocal image obtained by IG; (**h**) confocal image obtained by EG.

The annatto beads obtained by EG were 3.3 times larger (2291.040 ± 19.213 μm) than those obtained by IG (697.147 ± 20.395 μm) (Fig. 3a,b). In addition, the beads obtained by EG had a more homogeneous particle size than those obtained by IG. IG rendered a bimodal particle size population composed of a large one and smaller one. In addition, the surface of the beads was rough and did not show any difference with respect to the gelation type. The beads' cross-section (Fig. 3e,f) also showed that those obtained by IG have a more compact internal structure compared to those obtained by EG. Furthermore, the confocal microphotographs showed an anisotropic blue area where the encapsulated extract took place.

### FT-IR characterization of annatto extract

**Figure 4:**
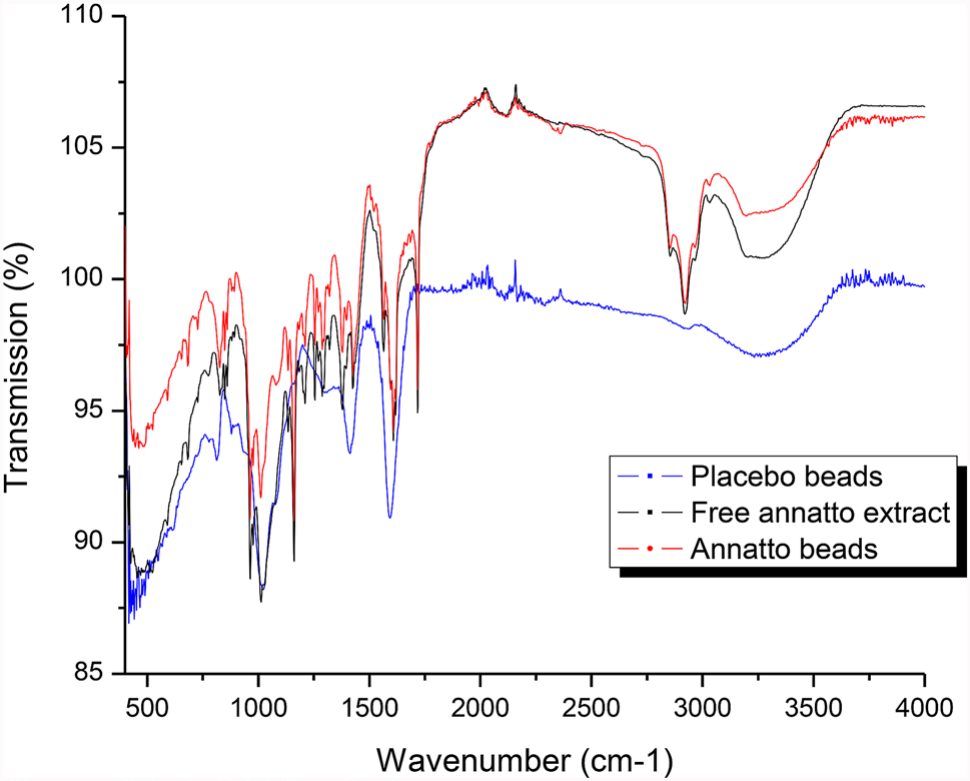
FT-IR spectrum obtained for free annatto extract, encapsulated by EG and blank (beads without extract obtained by EG).

Figure 4 shows the FT-IR spectra obtained for the samples. The placebo beads had a marked peak at 1029 cm^−1^ attributed to the presence of C–O–C of alginate. The bands at 1620 and 1395 cm^−1^ correspond to the symmetric and asymmetric vibrations of carboxylic groups, which are essential for the ionic gelation process^22^. In addition, the spectra of free annatto extract and annatto beads are observed at 3276 cm^−1^, which are attributed to the elastic vibrations of OH groups present in the alginate, bixin and polyphenol structures. The band at 1167 cm^−1^ and 1015 cm^−1^ represents the symmetric and asymmetric vibrations of C–O–C present in the bixin structure^23^.

### Release studies of bioactive compounds at different media conditions

**Figure 5:**
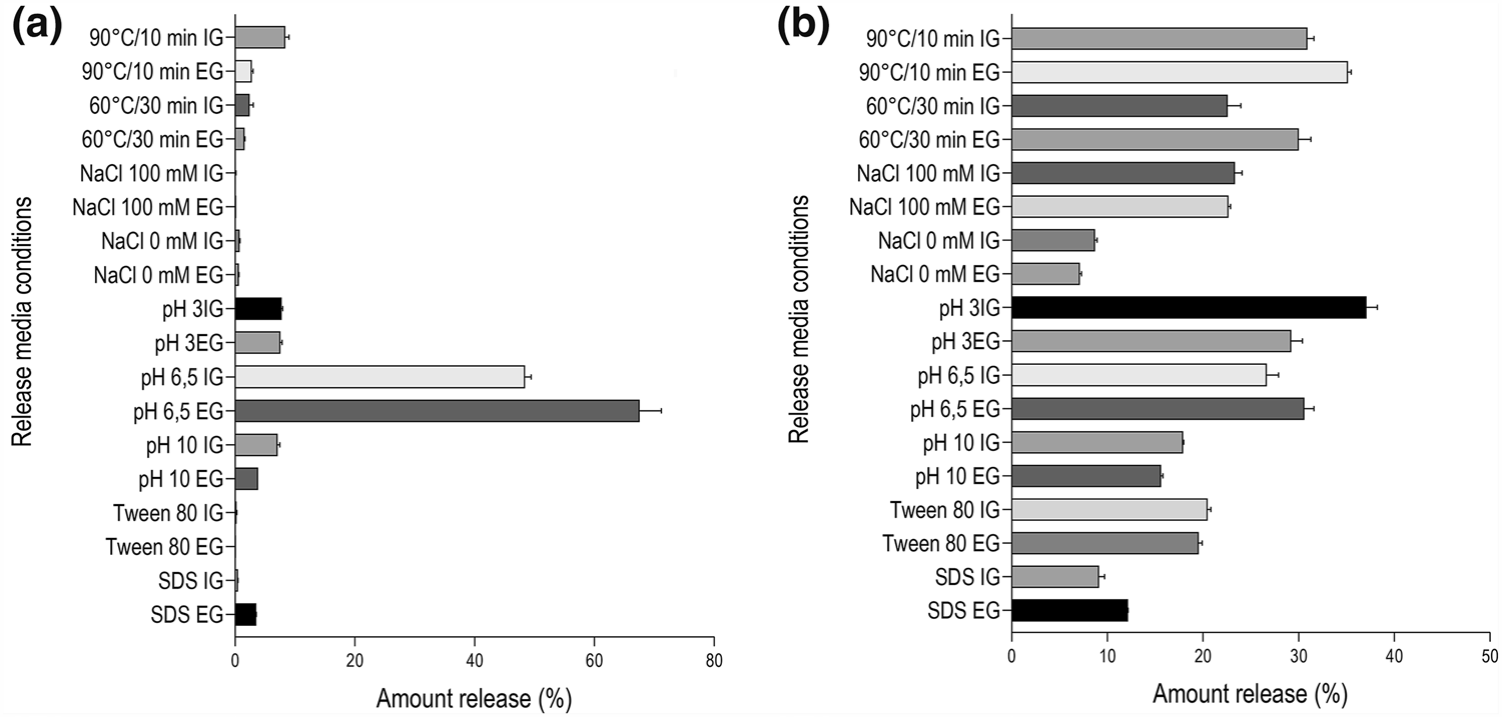
Effect of release media conditions on the release efficiency of BC from beads produced by EG and IG (**a**) bixin and (**b**) polyphenol compounds.

Figure 5 shows the release profiles of bixin and polyphenol compounds from beads suspended in a media with different pH, ionic strength, surfactants, and temperature conditions. Panel 5 a, shows the largest bixin release (67.56%, vs. 48.41%) at a pH of 6.5 for EG and IG, respectively (*p* = 0.000 and *p* = 0.198). At this pH, calcium ions compete with more complex ions such as phosphates changing the cross-linking magnitude with alginates^24^. Phosphate ions increase the hydrophobicity at neutral pH^25^, whereas, at acidic pH values, bixin (p*K*a 4.9) loses solubility in the media and precipitates, resulting in poor release in the phosphate buffer solution^23^. The release of bixin under other media conditions was below 10% due to the low bixin solubility in an aqueous medium. This is explained by an aliphatic conjugated double bonds structure and a carboxylic acid and a methyl ester group on each chain side^26^.

The polyphenol compounds release (Fig. 5b) was much lower (< 37%) than those obtained with bixin (67.56%). The highest release at 90 °C for beads obtained by EG (35.15%) followed by those obtained by IG (30.91%) and by EG at 60 °C (30%).

### Release kinetics of the bioactive compounds from bead produced by EG and IG

**Figure 6:**
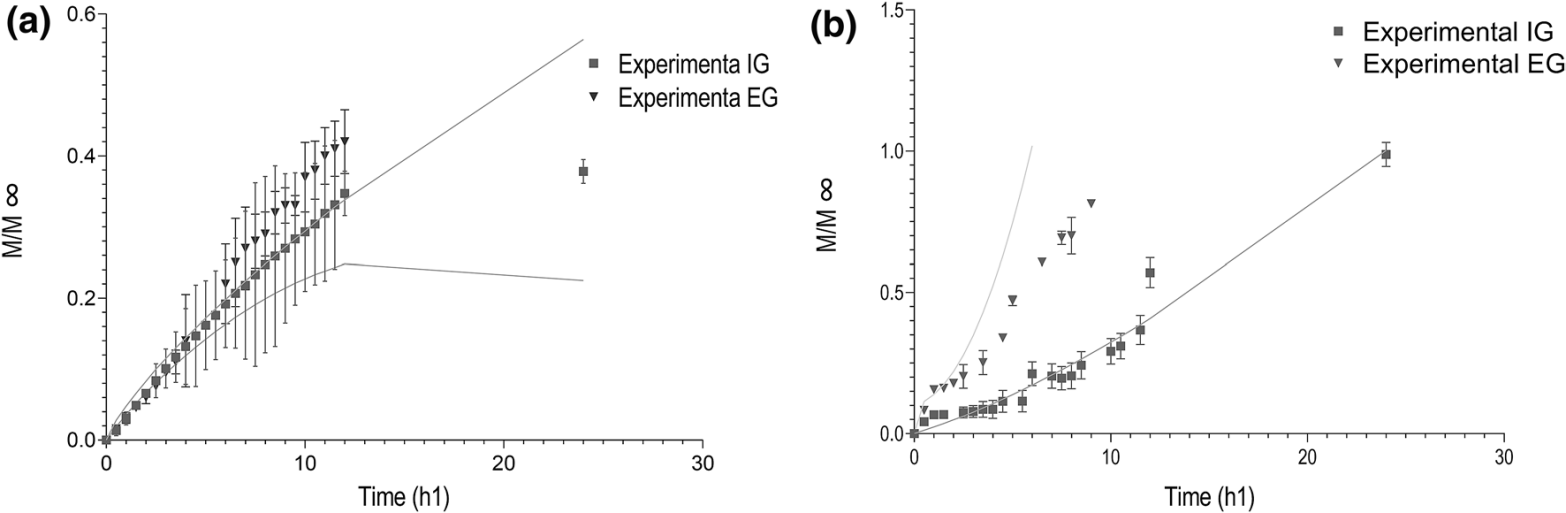
Kinetics release profile for (**a**) polyphenol compounds, and bixin (**b**) present in annatto extract encapsulated by EG (filled circles) and by IG (filled triangles) in a 0.1 M sodium phosphate buffer medium at a pH of 6.5, 25 °C and 210 rpm.

These studies were conducted at the conditions which give the greatest release. Thus is, at a pH of 6.5 in a medium containing 0.1 M Phosphate buffer. The fraction released with respect to time is shown in Fig. 6. Polyphenol compounds released from beads made by EG reach a fraction of 0.44 within 24 h, whereas those obtained by IG release a fraction of 0.37 of the polyphenol compounds (Fig. 6a). Conversely, the release kinetics of bixin was greater for beads made by EG (0.88) rather than IG (0.36) after 12 h (Fig. 6b).

Table 3 lists the parameters obtained from data fitting to the Peppas, Peppas–Burst, and Peppas–Sahlin models. In this case, the Peppas–Sahlin model showed the best fitting for the polyphenol compound independent of its technique. In addition, the value of the diffusion constant (k_1_) was greater than the value of the polymer relaxation constant (k_2_), indicating that a diffusional mechanism dominated the release of polyphenol compounds. Furthermore, the “m” values of 0.920 and 0.807 for beads obtained by EG and IG, indicates that most beads had a cylindrical shape and exhibited a purely Fickian diffusional behavior^27^. On the other hand, the Peppas–Sahlin model showed the best fit to the bixin experimental data; beads made by EG showed a k_2_ value slightly larger than k_1_, indicating that both mechanisms occurred for the release of bixin. Conversely, in beads made by IG, the diffusion constant (k_1_) was greater than the polymer relaxation constant (k_2_). In fact, the bixin k_1_ and k_2_ constant of EG were 100 times larger than those obtained for beads made by IG. In addition, the m values for EG ranged from 0.088 to 0.920, indicating a case II transport, whereas IG had values ranging from 0.703 and 0.807, indicating anomalous release mediated by both release mechanisms^27^.

**Table 3:** Release parameters obtained in a sodium phosphate buffer medium (0.1 M) at a pH of 6.5

BC	Sample	Model	k_1_	k_2_	a	n	m	r^2^	r^2^-aj
Polyphenol compounds	EG	Peppas	0.077			0.483		0.809	0.801
		Peppas–Burst	0.127		− 0.071	0.389		0.834	0.821
		Peppas–Sahlin	0.037	0.000			0.920	0.952	0.948
	IG	Peppas	0.087			0.456		0.851	0.845
		Peppas–Burst	0.135		− 0.066	0.374		0.872	0.862
		Peppas–Sahlin	0.048	0.000			0.807	0.969	0.967
Bixin	EG	Peppas	0.083			1.046		0.949	0.943
		Peppas–Burst	0.040		0.058	1.412		0.962	0.954
		Peppas–Sahlin	0.115	0.1400			0.088	0.983	0.979
	IG	Peppas	0.010			1.574		0.929	0.925
		Peppas–Burst	0.002		0.052	2.254		0.961	0.956
		Peppas–Sahlin	0.014	0.0100			0.703	0.959	0.955

*BC* bioactive compounds, *EG* external gelation, *IG* internal gelation.

## Discussion

### Encapsulation of annatto extract

The results from the experimental design for the optimization of the external ionic gelation mechanisms were in agreement with those obtained by Rezvanian and collaborators^28^, who studied the effect of different concentrations of CaCl_2_, concluding that calcium alginate films were formed at calcium levels as low as 0.5–1%. Additionally, the results found for annatto extract's encapsulation efficiency by the optimized EG and IG techniques are consistent with those found by other authors, who established that calcium alginate beads are suitable for the encapsulation of large molecules, especially hydrophobic materials with efficiencies larger than 90%^29^. In this case, it explains the high EE of bixin. On the contrary, the decrease in EE of bixin by IG might be explained by the high affinity of bixin molecules towards vegetable oil. In fact, bixin has a hydrocarbon chain with double conjugated bonds, which governs its non-polar nature and fat solubility^26^. On the other hand, polyphenols compounds showed EE below 95%, which was higher than those found by Belščak-Cvitanović and collaborators^30^. They obtained an EE of 73% for EG polyphenol compounds, whereas Moura and collaborators obtained values up to 88.5%^31^.

### Particle size and morphology

The beads' cross-section showed that those obtained by IG (Fig. 3f) have a more compact internal structure than those obtained by EG; the same phenomenon was observed by B. Lupo et al*.*^7^, who found that the gelation mechanism played a crucial role in the internal structure of the beads. These internal structures are related to the calcium-alginate reaction kinetics. In EG, the formation of the outer layer occurred at the beginning of the gelation process, decreasing the diffusion of calcium ions through the wall, leading to a low degree of crosslinking and, therefore more porous internal core. Most of the reported studies examine EG due to its simplicity. There are several studies that compare these two gelation mechanisms: For example, Chan and collaborators^32^, studied the impact of both techniques on the properties of calcium alginate films. They concluded that films obtained by EG had a less permeable surface due to the alginate chains, which were more cross-linked on the surface than within the core. They also established an optimal amount of cross-linking agent, since an excessive amount showed no statistically significant differences for the properties studied. On the other hand, Lupo et al.^7^ found significant differences in both techniques: particle size and texture were reduced when a high concentration of Ca^2+^ ions was employed. In addition, particles obtained by EG were harder and less flexible than those obtained by IG, whereas the latter demonstrated greater cohesiveness due to its smoother and more homogeneous structure independent of the amount of Ca^2+^ used.

#### Release studies of bioactive compounds

The release studies of bioactive compounds under different media conditions were similar to those obtained by Theaj and collaborators^33^. They reported that by increasing the media's temperature from 37 °C to 45 °C, the polyphenols release was increased from 73 to 90% in 48 h. In fact, a high temperature increases the media's kinetic energy, accelerating water penetration into the calcium alginate beads, promoting their swelling, and increasing the diffusion rate of the polyphenol compounds towards the media^18^. On the other hand, the ionic strength also had a statistically significant effect independent of the technique employed. The osmotic effect explains this phenomenon between media and beads, where the Na^+^ ions in the media diffuse across the calcium alginate beads replacing Ca^2+^ ions, hence disrupting the bead structure by increasing the release rate^18^. In addition, polyphenol compounds release between 26.67 and 37.09% was favored at a pH of 3.0 and 6.5 independent of the technique employed. Other authors report the highest release of the polyphenolic compounds such as procyanidin, catechin and epicatechin at a pH of 1.4^34^. In fact, the release kinetics from calcium alginate beads depended on factors such as hardness, porosity, solubility, and affinity towards the media. In addition, the conditions of the release media affect the degree of swelling and erosion. Generally, most polyphenol compounds are hydrophilic and hence are released by diffusion mechanisms, whereas hydrophobic compounds such as bixin are mainly released by erosion^35^. A diffusional mechanism dominated the release of polyphenol compounds. This phenomenon was explained by the migration of some water-soluble polyphenol compound from the core to the outer surface during the freeze-drying process. Once the beads were in contact with the release media, many of these compounds on the surface were released at a higher diffusion rate^36,36^. These results are similar to those found by Singh et al.^38^, who reported an anomalous kinetics release for fungicide-alginate beads reinforced with starch and clay. However, the work carried out by Arriola et al.^36^ encapsulating stevia extract adjusted to the Peppas–Burst model (r^2^ = 0.99) revealed a diffusion release mainly due to the low molecular weight of the polyphenol compounds and their high solubility in aqueous systems that facilitated their diffusion throughout the porous structure even before swelling or erosion occurred^37^.

Bera et al. studied the release of carotenoids from hydrogel particles in aqueous media^39^, and found that bacterial canthaxanthin released from alginate and chitosan hydrogels was larger in neutral media than in acidic media, and follows an anomalous release with n values of 0.723 and 0.778, respectively. The Peppas model parameters, with r^2^-adjusted values of 0.943 for beads obtained by EG and 0.925 for IG, and n values > 0.85 for both samples, indicate a case II super relaxation associated with the vitreous stress of hydrophilic polymers that swell in water^27,40,41^. In conclusion, EG mechanism presented the highest process yield and the encapsulation efficiencies of the bioactive compounds were higher than those obtained by IG. However, the beads obtained by GE at the optimal conditions, presented a large particle size, unsuitable to use in food or pharmaceutical applications, so it is recommended for future research to study how to reduce the particle size used by emerging technologies such as electrospray.

## References

[1] Quintero-Quiroz J (2019). Optimization of the microwave-assisted extraction process of bioactive compounds from annatto seeds (*Bixa**orellana* L.). Antioxidants.

[2] Cuong TV, Thoa NT (2017). Effects of partial replacement of nitrite by annatto (*Bixa**orellana* L.) seed powder on the properties of pork sausages. J. Sci. Technol..

[3] Shahid-ul-Islam, Luqman, J. R., Faqeer, M. (2016). Phytochemistry, biological activities and potential of annatto in natural colorant production for industrial applications—A review. J. Adv. Res. **7**, 499–514. 10.1016/j.jare.2015.11.002 PMC4856788 27222755

[4] Butnariu M (2016). Methods of analysis (extraction, separation, identification and quantification) of carotenoids from natural products. J. Ecosyst. Ecogr..

[5] Quintero-Quiroz J, Naranjo-Duran AM, Silva-Garcia M, Ciro-Gomez GL, Rojas-Camargo JJ (2019). Ultrasound-assisted extraction of bioactive compounds from annatto seeds, evaluation of their antimicrobial and antioxidant activity, and identification of main compounds by LC/ESI-MS analysis. Int. J. Food Sci..

[6] Medina-Flores D (2016). Antibacterial activity of *Bixa**orellana* L. (achiote) against *Streptococcus**mutans* and *Streptococcus**sanguinis*. Asian Pac. J. Trop. Biomed..

[7] Lupo B, Maestro A, Gutierrez JM, Gonzalez C (2015). Characterization of alginate beads with encapsulated cocoa extract to prepare functional food: Comparison of two gelation mechanisms. Food Hydrocoll..

[8] Naranjo-Duran A. M., Quintero-Quiroz, J. & Ciro-Gómez G. L. Optimización del proceso de lixiviación de los compuestos bioactivos de las semillas de *Bixa orellana* L. (annato). Rev Cuba Plantas Med. **22** (2017).

[9] Gazori T (2009). Evaluation of alginate/chitosan nanoparticles as antisense delivery vector: Formulation, optimization and in vitro characterization. Carbohydr. Polym..

[10] Chan E-S (2011). Preparation of Ca-alginate beads containing high oil content: Influence of process variables on encapsulation efficiency and bead properties. Carbohydr. Polym..

[11] Nunes MA (2018). Olive pomace as a valuable source of bioactive compounds: A study regarding its lipid- and water-soluble components. Sci Total Environ..

[12] Mínguez-Mosquera MI, Hornero-Méndez D, Pérez-Gálvez A, Hurst WJ (2008). Carotenoids and provitamin A. Functional foods. Methods of analysis for functional food.

[13] FAO/WHO. *Compendium of Food Additive Specifications; Joint FAO/WHO Expert Committee on FoodAdditives* vol. 4 (2006).

[14] FAO. *Total Colouring Matters Content* vol. 4.

[15] Hosseini SM (2013). Incorporation of essential oil in alginate microparticles by multiple emulsion/ionic gelation process. Int. J. Biol. Macromol..

[16] Londoño C, Rojas J (2017). Effect of different production variables on the physical properties of pellets prepared by extrusion-spheronization using a multivariate analysis. Pharm. Sci..

[17] Belščak-Cvitanović A (2016). Emulsion templated microencapsulation of dandelion (*Taraxacum**officinale* L.) polyphenols and β-carotene by ionotropic gelation of alginate and pectin. Food Hydrocolloids.

[18] Tan PY (2017). Effects of environmental stresses and in vitro digestion on the release of tocotrienols encapsulated within chitosan-alginate microcapsules. J. Agric. Food Chem..

[19] Prataa AS, Zanin MHA, Maria I, Ré MI, Grosso CRF (2008). Release properties of chemical and enzymatic crosslinked gelatin-gum Arabic microparticles containing a fluorescent probe plus vetiver essential oil. Colloids Surf. B Biointerfaces.

[20] Sun X, Cameron RG, Bai J (2019). Microencapsulation and antimicrobial activity of carvacrol in a pectin-alginate matrix. Food Hydrocoll..

[21] Yu C-Y (2009). Composite microparticle drug delivery systems based on chitosan, alginate and pectin with improved pH-sensitive drug release property. Colloids Surfaces B Biointerfaces.

[22] Das RK, Kasoju N, Bora U (2010). Encapsulation of curcumin in alginate-chitosan-pluronic composite nanoparticles for delivery to cancer cells. Nanomed. Nanotechnol. Biol. Med..

[23] Rahmalia W, Fabre JF, Mouloungui Z (2015). Effects of cyclohexane/acetone ratio on bixin extraction yield by accelerated solvent extraction method. Procedia Chem..

[24] Paques JP, Sagis LMC (2015). Alginate nanospheres prepared by internal or external gelation with nanoparticles. Microencapsulation and microspheres for food applications.

[25] Jahangiri A (2019). Incorporation of bixin in aqueous media: Self-formulation with sorbitol ester of norbixin. Food Chem..

[26] Jahangiri A (2018). Hydrophilization of bixin by lipase-catalyzed transesterification with sorbitol. Food Chem..

[27] Peppas NA, Sahlin JJ (1989). A simple equation for the description of solute release. III. Coupling of diffusion and relaxation. Int. J. Pharm..

[28] Rezvanian M, Ahmad N, Mohd MCI, Ng SF (2017). Optimization, characterization, and in vitro assessment of alginate-pectin ionic cross-linked hydrogel film for wound dressing applications. Int. J. Biol. Macromol..

[29] Leong JY (2016). Advances in fabricating spherical alginate hydrogels with controlled particle designs by ionotropic gelation as encapsulation systems. Particuology.

[30] Belščak-Cvitanović A (2019). Encapsulation templated approach to valorization of cocoa husk, poppy and hemp macrostructural and bioactive constituents. Ind. Crops Prod..

[31] Aguirre-Calvo TR, Perullini M, Santagapita PR (2018). Encapsulation of betacyanins and polyphenols extracted from leaves and stems of beetroot in Ca(II)-alginate beads: A structural study. J. Food Eng..

[32] Chan LW, Lee HY, Heng PWS (2006). Mechanisms of external and internal gelation and their impact on the functions of alginate as a coat and delivery system. Carbohydr. Polym..

[33] Theaj R, Upputuri P (2019). Mathematical modeling and release kinetics of green tea polyphenols released from casein nanoparticles. Iran. J. Pharm. Res..

[34] Lavelli V, Sri Harsha PSC (2019). Microencapsulation of grape skin phenolics for pH controlled release of antiglycation agents. Food Res. Int..

[35] Costa P, Lobo JMS (2001). Modelling and comparison of dissolution profiles. Eur. J. Pharm. Sci..

[36] Aceval-Arriola N, de Medeiros PM, Prudencio ES, Carmen Maria Olivera-Müller CM (2016). Encapsulation of aqueous leaf extract of *Stevia**rebaudiana* Bertoni with sodium alginate and its impact on phenolic content. Food Biosci..

[37] Huang X, Brazel CS (2001). On the importance and mechanisms of burst release in matrix-controlled drug delivery systems. J. Control Release.

[38] Singh B, Sharma DK, Kumar R, Gupta A (2009). Controlled release of the fungicide thiram from starch–alginate–clay based formulation. Appl. Clay Sci..

[39] Bera S, Dutta D (2017). Encapsulation and release of a bacterial carotenoid from hydrogel matrix: Characterization, kinetics and antioxidant study. Eng. Life Sci..

[40] Siepmann J, Peppas N (2001). Modeling of drug release from delivery systems based on hydroxypropyl methylcellulose (HPMC). Adv. Drug Deliv. Rev..

[41] Ritger PL, Peppas NA (1897). A simple equation for description of solute release II. Fickian and anomalous release from swellable devices. J. Control Release..

